# A comprehensive multimodal dataset for contactless lip reading and acoustic analysis

**DOI:** 10.1038/s41597-023-02793-w

**Published:** 2023-12-13

**Authors:** Yao Ge, Chong Tang, Haobo Li, Zikang Chen, Jingyan Wang, Wenda Li, Jonathan Cooper, Kevin Chetty, Daniele Faccio, Muhammad Imran, Qammer H. Abbasi

**Affiliations:** 1 James Watt School of Engineering, University of Glasgow, Glasgow, G12 8QQ UK; 2 Department of Security and Crime Science, University College London, London, WC1E 6BT UK; 3 School of Physics & Astronomy, University of Glasgow, Glasgow, G12 8QQ UK; 4 School of Science and Engineering, University of Dundee, Dundee, DD1 4HN UK

**Keywords:** Electrical and electronic engineering, Design, synthesis and processing, Biomedical engineering

## Abstract

Small-scale motion detection using non-invasive remote sensing techniques has recently garnered significant interest in the field of speech recognition. Our dataset paper aims to facilitate the enhancement and restoration of speech information from diverse data sources for speakers. In this paper, we introduce a novel multimodal dataset based on Radio Frequency, visual, text, audio, laser and lip landmark information, also called RVTALL. Specifically, the dataset consists of 7.5 *GHz* Channel Impulse Response (CIR) data from ultra-wideband (UWB) radars, 77 *GHz* frequency modulated continuous wave (FMCW) data from millimeter wave (mmWave) radar, visual and audio information, lip landmarks and laser data, offering a unique multimodal approach to speech recognition research. Meanwhile, a depth camera is adopted to record the landmarks of the subject’s lip and voice. Approximately 400 minutes of annotated speech profiles are provided, which are collected from 20 participants speaking 5 vowels, 15 words, and 16 sentences. The dataset has been validated and has potential for the investigation of lip reading and multimodal speech recognition.

## Background & Summary

In general speech recognition tasks, acoustic information from microphones is the main source for analyzing the verbal communication of humans^1^. The speech process is not just a means of conveying linguistic information, which can also provide valuable insight into the speaker’s characteristics such as gender, age, social and regional origin, health, emotional state, and in some cases even their identity. Recently, the automatic speech recognition (ASR) technique has already matured and been marketed^2^. In addition to sound signals, the series of physiological processes that produce sound, such as lip movement, vocal cord vibration, and head movement, also retain semantic and speaker information to some extent. On the other hand, there are two main limitations in specific environments that only audio information can not perfectly work for ASR: silent speech recognition (SSR) and multiple speakers environments. Both issues can be solved if considering the speaker physics properties and will be explained in following paragraphs.

First, SSR can be considered a significant branch of speech recognition that provides understandable and enhancing communication methods to assist patients with severe speech disorders. In recent years, research in silent speech recognition has explored a variety of approaches, including wearable sensors, radar-based systems, and other non-invasive techniques, to address the challenges of capturing and processing speech-related information. The contactable methods mainly focus on detecting brain and muscle activity with electroencephalogram (EEG) sensor, articulator movements headset and other types of implantable sensors^3^. However, contact based methods are highly dependent on wearable and implant sensors, which is dedicated to patients but does not collect a large dataset from a normal person. Meanwhile, users should consider the potential health risk of contactable devices. For voice disorder and other patients who maintain the capability to control the vibration of vocal folds and face muscles, non-invasive SSR has the potential to improve their quality of life compared to electronic sensors.

In addition, in scenarios with multiple speakers, the microphone captures the sounds from the surroundings without distinguishing the person’s identity, which seriously lowers the accuracy of speech recognition. This issue is similar to the cocktail party effect^4^, which is a phenomenon in which an individual can focus on one conversation despite being surrounded by several other simultaneous conversations. The effect is mainly attributed to the brain’s ability to process auditory frequency and highlight certain sounds, allowing the individual to focus in on the source of interest without being easily distracted. However, it is a challenge to separate different sources only using acoustic data. In this case, additional radar or laser devices can assist the model in distinguishing the audio according to the physical information. For example, the proposed work^5^ combined the audio and radar signals to filter after added noise. And Secondly, the voice information including tone and speaking habits of individual contains a variety of personal data that can be used to create a unique voice fingerprint, such as speaking habits and intonation. This will cause a risk of sensitive data leakage, as the voice fingerprint could be used for identification. For the wireless sensing side-based algorithm, vocal folds vibration focuses only on the tone of speech, which does not include privacy information. Third, previous speech recognition research has focused mainly on visual-based mouth movements, posing a risk of lack of privacy and overlooking internal mouth movements.

In this paper, we proposed a dataset of human speech by collecting data from multiple sensors information while people are speaking specific corpus. The contributions of our dataset are concluded in following points:

1. In this work, we present a novel dataset that incorporates multiple modalities for silent speech recognition, including ultra-wideband (UWB) radars, millimeter wave (mmWave) radar, and depth camera data, which we believe will be a valuable resource for researchers in the field. The dataset is expected to reduce the labour for researchers who expect to work on SSR from wireless signals or enhancing audio signals.
2. Our system takes into account the physical movements of all parts of the head during human speech, including mouth movements and vibrations of the vocal cord, which is illustrated in Fig. 1.

**Fig. 1:**
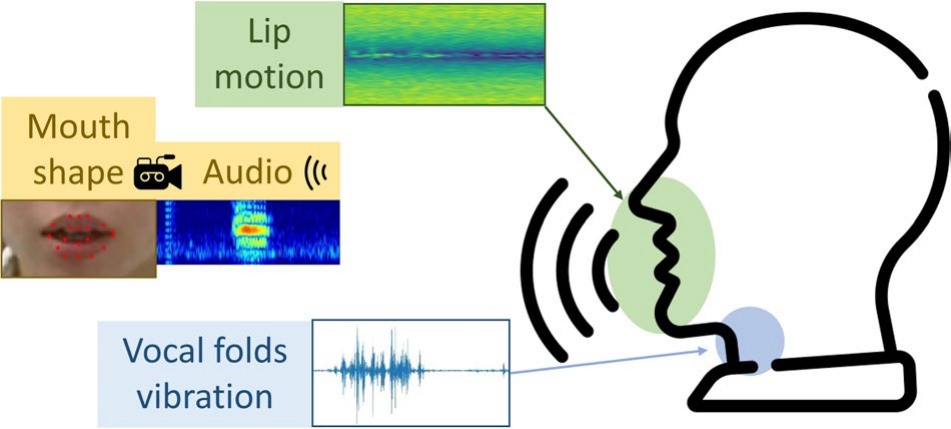
Schematic diagram of the Multimodal signals.
3. The diverse range of modalities in our dataset offers ample opportunities for conducting research in the field of speech recognition. The range contains but is not limited to the following application: radar-based vowels and words classification, speaker identification, speech enhancement in noisy environment, radar-based lip reconstruction, etc.

## Methods

Firstly, we conducted a literature review to establish the necessary sensors and experimental setup for radar-based speech recognition, given the absence of a standard and corpus. Meanwhile, we demonstrate the availability of all the sensors we adopted and then establish our data collection approach, referencing previous work.

### Literature survey of radar-enabled speech recognition

There are various kinds of sensors has been adopted for speech research: UWB, mmWave radar and laser speckle detector^6^. For SSR task, the work of UWB demonstrated the lip reading work with the vowels of [æ], [i], [ә], [ɔ:], [u:] and static scenario, with a face mask. The result of 95% approves that the mouth motion produces informative signals for UWB sensing^7^. FMCW radar is also an optional choice which has been proven in the result of the paper^8^. The mentioned work adopts point clouds of human mouth while speaking as data feature for classification work of 13 words with 4 speakers. It gains 88% accuracy using Linear Regression classifiers. To expand the work and exploit more possibilities, we added sentences for data collection regarding the reference. Besides, mmWave FMCW radar has been used for speech enhancement in the published works^5,6^. These two researches have distinct focus directions: the paper^5^ considered the distance coefficient for radar signals and successfully make speech enhancement system implementation in 7 meters; and works^6,9^ on audio separation of multiple speakers with radar-based spatial information. For laser-related information, paper^10^ proposed a remote measurement technique for healthy individuals that involves capturing the reflected laser speckle from the surface of the neck skin. This system is capable of capturing the micro-vibration on the surface of the neck produced by blood pressure, which can also be adopted for the extraction of voice signals without audio signals through detecting the vibration caused by throat. Inspired by mentioned works, we decided to adopt radar sensors of FMCW radar and UWB radar, laser speckle detection system, and Kinect camera for mouth skeleton stream and speech voice as the source of our multimodal dataset.

Furthermore, we conclude the dataset of those multimodal based speech recognition works in Table 1. To the best of our knowledge, most open-access datasets in speech recognition focus on audiovisual topics instead of considering radio signals. Although there has been research talking about wireless signals based speech processing, it is difficult to get the dataset from authors. Therefore, our core contribution is establishing a contactless based speech dataset for research on combination of audio signals and physically vibration from wireless signal.

**Table 1:** Dataset review of multimodal based speech recognition works.

	Dataset size	Dataset usage	Multimodal type	Open access of dataset provided
RAVDESS^21^	7356 samples	emotion recognition	visual video stream, voice	Yes
SpeakingFaces^22^	13000 samples	biometric authentication, speech recognition	visual video stream, thermal video stream, voice	Yes
UltraSE^23^	8000 samples of 5 seconds speech	speech enhancement	ultrasound signal, voice	Not found
RadioSES^24^	5700 sentences	speech separation and enhancement	FMCW radar signal, voice	Not found
Speckle detection^25^	1000 sample of speech	speckle noise removal	laser signal, voice	Not found
Electromyogram^26^	660 words	silent speech recognition	high-density surface electromyogram	Not found
SSR^20^	few samples for validation	speech enhancement	laser signals	Not found
AV-corpus^27^	34000 sentences	speech recognition	audio and video	Yes
IEMOCAP^28^	10039 samples of 4.5 seconds speech	emotion recognition	audio, video, face and hand markers	Yes
TaL^29^	18221 samples	speech recognition	audio, video stream, ultra sound image	Yes
Ours	6000 samples (including vowels, words and sentences)	silent speech recognition, speech separation, speech enhancement	FMCW radar signal, UWB radar signal, mouth skeleton, laser signal, and voice	Yes

### Data acquisition scheme

The overall data collection system was organized by four laptops and four types of sensors: Microsoft Kinect V2 for audio and video including mouth landmark, X4M03 UWB radar from NOVELDA, AWR2243 mmWave radar from Texas Instrument, and laser measurement system for physical vibration of human speech. The selection of devices is referred from mentioned previous research. To keep the data synchronised with different sensors, we used the TCP/IP connection to control the distinct host laptops with the same network time protocol (NTP) for recording the time stamp while data collection. A multi-threaded control script has been developed and employed that automatically initiated and terminated data recording scripts, minimizing data recording latency to the greatest extent. Once we run the script on a master laptop, the master will send the commands to the other three sockets in series. The mean delay from the master to sockets of other devices is around 80 ms, which is considered in our post-synchronization processing. Furthermore, we employed expert supervision and manual calibration to ensure time synchronization across different sensors. We calibrated the devices, monitored the data collection process, and made necessary adjustments throughout the entire data collection. Considering the potential research for speech recognition, we designed three data collection schemes shown in the following. The adopted corpus is recorded in an additional folder in our dataset.

- Single person speech of vowels, words and sentences.
- Dual-person speech simultaneously of complex sentences.
- Single person speech of vowels, words and sentences with different distance from radar to speakers.

**Fig. 2:**
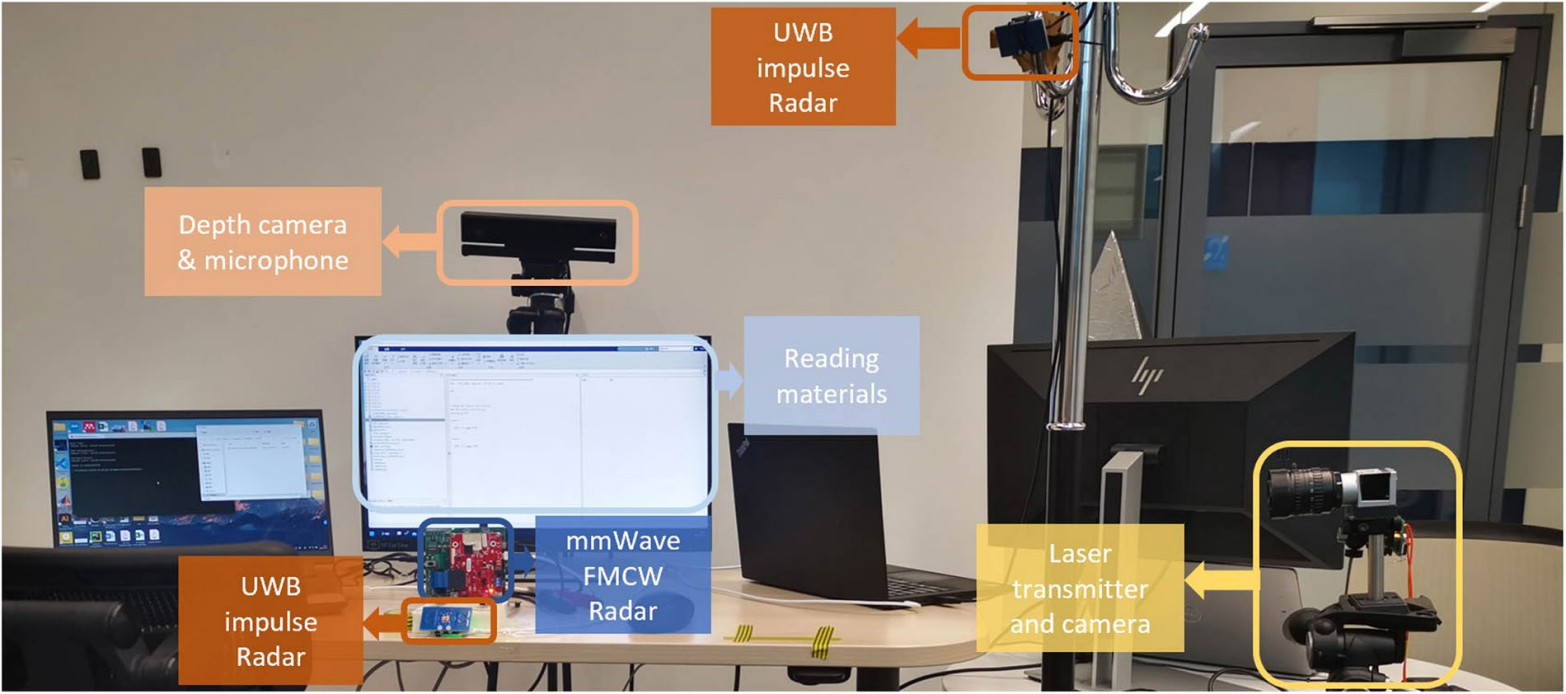
Data collection setup with device label in the real scenario.

The details of data collection from specific sensor are demonstrated below, with experiment setup shown in Fig. 2.

### Speech voice

We used Kinect v2 for collecting vocalised speech. With the enable Kinect v2 to collect the accurate acoustic information. The sample rate of audio data is 16 *kHz*, and bit depth is 16-bit. The frequency range of recording audio is up to 8 *kHz*, that can cover the frequency range of human voice.

### Mouth skeletal points

The Kinect v2 is also used in collecting the facial landmark information. A RGB camera and an infrared camera are intergrated in kinect v2. By measuring the time of flight (ToF) using the IR camera Kinect can get the depth image. Meanwhile, we use the lip recognition method proposed in paper^11^ for extraction of the lip skeleton, which is provided as part of our dataset.

### IR-UWB radar

Like Wi-Fi and Bluetooth, UWB is a short-range wireless communication protocol. The UWB was defined as the wireless transmission system of which the bandwidth exceeds 500 *MHz*, and each transmit pulse of this communication system can occupy at least 500 *MHz* bandwidth. Instead of modulating with a carrier wave, IR-UWB relies on nanosecond (ns) to picosecond (ps) non-sinusoidal narrow impulse radio signals to transmit data. The time-based modulation technology increases transmission speed and reduces power consumption. For speech recognition, the UWB system has the following advantages:

1. Strong anti-interference ability: From the RF mechanism, the pulse wave emitted by UWB is more resistant to interference than the continuous electromagnetic waves in short range. Specifically, the permitted work frequency band of UWB is from 3 *GHz* to 10 *GHz*, which suffers less disturbance from the general 2.4 *GHz* WiFi system and other telecommunication signals.
2. The protocol yielded positive results, resulting in a reduction in power consumption for short-range communication applications. The transmit power of the UWB transmitters was found to be typically less than 1 mW, which extended the system’s operating time and minimized electromagnetic wave radiation to the human body.

After careful consideration of cost and feasibility, we have selected the XeThru X4M03, an IR-UWB radar system on chip, as our UWB radar. The UWB RF specifications of this radar have been approved by ETSI (European Telecommunications Standards Institute) in Europe, and FCC (Federal Communications Commission) in the USA for commercial use in human living circumstances^12^.This device is a highly reliable sensor that is capable of detecting objects at a range of up to 10 meters. It is also capable of detecting objects in a wide range of angles, up to 180 degrees. This radar system has been adopted in a variety of research projects, ranging from human vital sign detection^13,14^ to activity recognition^15^.

For pulsed radars, the distance between the radar and the target can be determined by $R=\frac{c\ast \Delta T}{2}$, where *c* represents microwave speed, Δ*T* represents the round-trip time of a single pulse, called time of flight (ToF). The signals of IR-UWB can be represented in Eq. 1, where the *τ* represents the ToF of signals impulses in fast-time range, *t* represents receiving time of frame in slow-time domain, *N*_*d*_ is the index of the dynamic path, ${a}_{i}(\tau ,t)$ represents the complex attenuation factor of the *i*^*th*^ path; ${e}^{-j2\pi \frac{d(t)}{\lambda }}$ represents the phase change of *i*^*th*^ path; *d*_*i*_(*t*) and *d*_*a*_(*t*) are the static length and vibrating length of *i*^*th*^ path. *λ* represents the wavelength of the UWB signal.

1 $${\bf{s}}(\tau ,t)=\mathop{\sum }\limits_{i=1}^{{N}_{d}}{a}_{i}(\tau ,t){e}^{-j2\pi \frac{({d}_{i}(t)+{d}_{a}(\tau ))}{\lambda }}$$

**Fig. 3:**
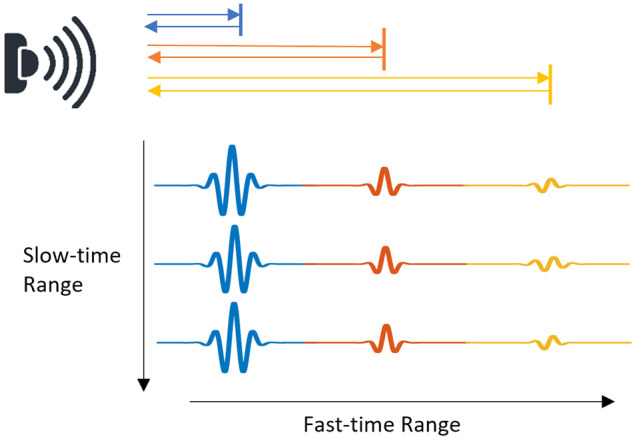
IR-UWB impulse signals format.

The format of the received signals consists of a fixed set of bins, determined by the timing of the transmitted pulses. These bins are indexed by fast-time and slow-time dimensions, as illustrated in Fig. 3. Fast-time and slow-time are two dimensions that are used to describe the format of the received signals in a UWB radar system. Fast-time is the time it takes for the radar to transmit a pulse and receive the reflected signal. Slow-time is the time it takes for the radar to transmit multiple pulses and receive the reflected signals. The fast-time and slow-time dimensions are used to determine the format of the received signals, which is a fixed set of bins. These bins are used to store the information about the objects detected by the radar. The fast-time and slow-time dimensions are also used to determine the range of objects detected by the radar. By using the fast-time and slow-time dimensions, the UWB radar system can accurately detect objects in a wide range of distances. This makes the UWB radar system an ideal choice for a variety of applications, including human vital sign detection and activity recognition.

For data collection, we adopted Moduleconnector API which is supported by the radar on Windows MATLAB 2021b. The detailed parameters of radar are listed in the Table 2.

**Table 2:** Radar parameter setup of UWB X4M03 and TI AWR2243.

(a) UWB radar setup	
Parameter	Value
Center frequency	8.745 GHz
Sampling frequency	23.328 GHz
Frame rate	300 Hz
Bandwidth	1.5 GHz
Number of antennas	1 Tx and 1 Rx
**(b) mmWave radar setup**	
Center frequency	79 GHz
ADC Sampling frequency	10 msps
ADC samples	512
Frame rate	1018 Hz
Ramp End Time	60 us
Bandwidth	3.8 GHz
Range resolution	3.95 cm

### mmWave FMCW radar

**Fig. 4:**
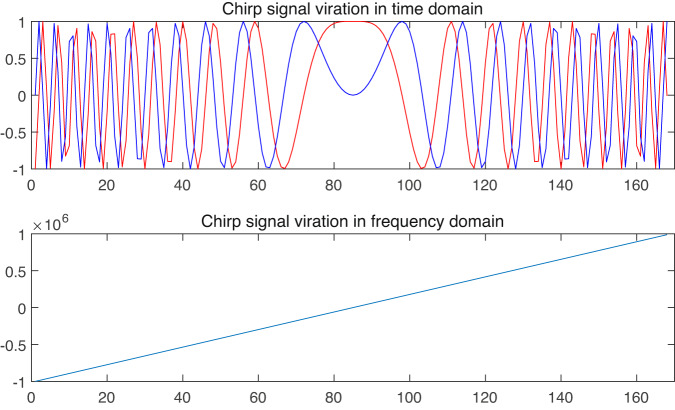
The plot of a single mmWave FMCW radar chirp in time and frequency domain.

Although the IR-UWB radar is able to capture the vibration of sub-centimeter motion. The angle resolution is limited by the number of antennas. Meanwhile, for comparison of speech recognition performance using different modulation-based radars, we added one commercial off-the-shelf (COTS) 77 *GHz* FMCW radar, AWR2243, for data collection. This high frequency enables radar signals to capture motion in millimeter, that can be used for both lip motion and vocal folds detection. A FMCW radar chirp signals can be illustrated as Fig. 4.

Unlike IR-UWB radars that measure distance using the ToF of instantaneous impulses, FMCW radars rely on the difference in frequency between the transmitted and received signals from a linear variation of signals frequency. In other words, Doppler effect of moving target can be explored from difference between transmission frequency (*f*_*t*_) and shifted frequency (*f*_*s*_). The formula shows Doppler velocity of target relative to radar: $v=\frac{c\left({f}_{s}-{f}_{t}\right)}{2ft}$.

Except for modulation methods, AWR2243 radar contains 4 receive antennas and 3 transmit antennas, which is possible to adopt angle of arrival (AoA) for research in horizontal plane. In our experiment, we adopted 4 receive and 1 transmit antennas to increase the sample rate. By adopting 4 channels at a snapshot, we can gain the angular resolution of 30 degree. In data collection, we utilised mmWaveStudio API on Windows with the configuration file. The parameters were set to the value shown in Table 2. The frequency response reflected by the radar signals was of paramount interest, particularly given the average fundamental frequency range of the vocal folds, which spanned from 85 *Hz* to 255 *Hz*^16^. Aligning with the Nyquist criterion, we adjusted our radar frame rate to 1018 *Hz*, ensuring the sampling rate exceeds twice the highest frequency present in the signal.

### Laser-speckle system

The laser measurement system consists of a 532 nm green laser diode (DJ532-40, Thorlabs) as transmitter and a high-speed CMOS camera from Basler as receiver, where the laser diode emits a laser beam pointing to the face outline of the testing subject and the camera captures the reflected laser speckle patterns. Both transmitter and receiver are fixed on a 1.2 m tripod, and the camera is connected with a laptop via an USB 3.0 cable for powering and data transferring. The green laser diode has a distance of approximately 1 m to the participants, it will produce an illumination spot of around 5 mm diameter on the human skin by considering the beam divergence. For the laser safety, laser power exposed on human skin is controlled to be less than 0.5 mw (CLASS 1), therefore it is safe for long-term eye and skin exposure. The focal length and f-stop of the camera objective are set as 25 mm and 0.95, respectively, allowing the camera system to detect the laser speckle from a very close range (0.1 m) to a relatively far range (up to 3 m). Furthermore, the size of the region of interest (ROI) window is chosen as 128 × 128 pixels, and the camera exposure time is set as 600 µs, shown in Table 3. The laser and camera are carefully aligned before the experiments to ensure that the selected ROI includes the movements of speckles. For each measurement, the collected data is in a format of *W* × *H* × *N*, where W and H represent the width and height of the ROI, respectively, and the N equals to the number of frames within the measurement period. In our case, N is correlated with the sampling frequency of the CMOS camera, which is set as 1.47 *kHz*.

**Table 3:** Parameter setup of Laser speckle detection system.

(a) Laser transmitter setup	
Parameter	Value
Power	0.5 mw
Wavelength	532 nm
Working Mode	continuous wave
Beam Divergence	12 mrad
Operating Current	330 mA
Operating Voltage	1.9 V
**(b) Camera setup**	
FPS	1470
Gain	12
Exposure Time	600 us
ROI Size	128×128 pixel

### Participant

There are 20 volunteers contributing to our experiment, who come from different country regions including Europe, China, and Pakistan. The volunteer information sheet is listed in Table 4. Our dataset for speech recognition presents both opportunities for generalization and challenges due to the volunteers’ diverse backgrounds, resulting in distinct accents. To the issue bring from body size, we adopted an adjustable table for subjects that can keep the relative distance same between the head of speaker and different sensors. The characteristic of accents and speech habits can be extracted from our dataset with lip motion, vocal folds vibration, and audio, which has potential for related multimodal ASR research.

**Table 4:** Volunteer information sheet.

Volunteer index	Age	Gender	Native language	Education
1	25	Male	Chinese	BEng
2	25	Female	Chinese	BEng
3	24	Male	Chinese	MSc
4	25	Male	Chinese	BEng
5	22	Male	Chinese	BEng
6	25	Male	Chinese	BEng
7	31	Male	Arabic	MSc
8	24	Female	Chinese	BEng
9	30	Male	English	PhD
10	25	Male	Chinese	MSc
11	24	Male	Chinese	BEng
12	23	Male	Chinese	BEng
13	24	Female	Chinese	BEng
14	34	Female	Arabic	MSc
15	25	Female	English	BEng
16	30	Female	Arabic	MSc
17	25	Male	Chinese	BEng
18	25	Female	Chinese	MSc
19	24	Male	English	PhD
20	31	Male	Arabic	PhD

Meanwhile, all participants were informed about the purpose of the study, the implications of identifying information, and what was expected of them. They agreed to the open publication of identifiable information including voice signal in released dataset. Experiment consent forms were obtained from each participant prior to the experiments. The entire dataset collection project was ethically approved by the University of Glasgow College of science and engineering (approval no: 300210309).

### Setup of data collection

**Fig. 5:**
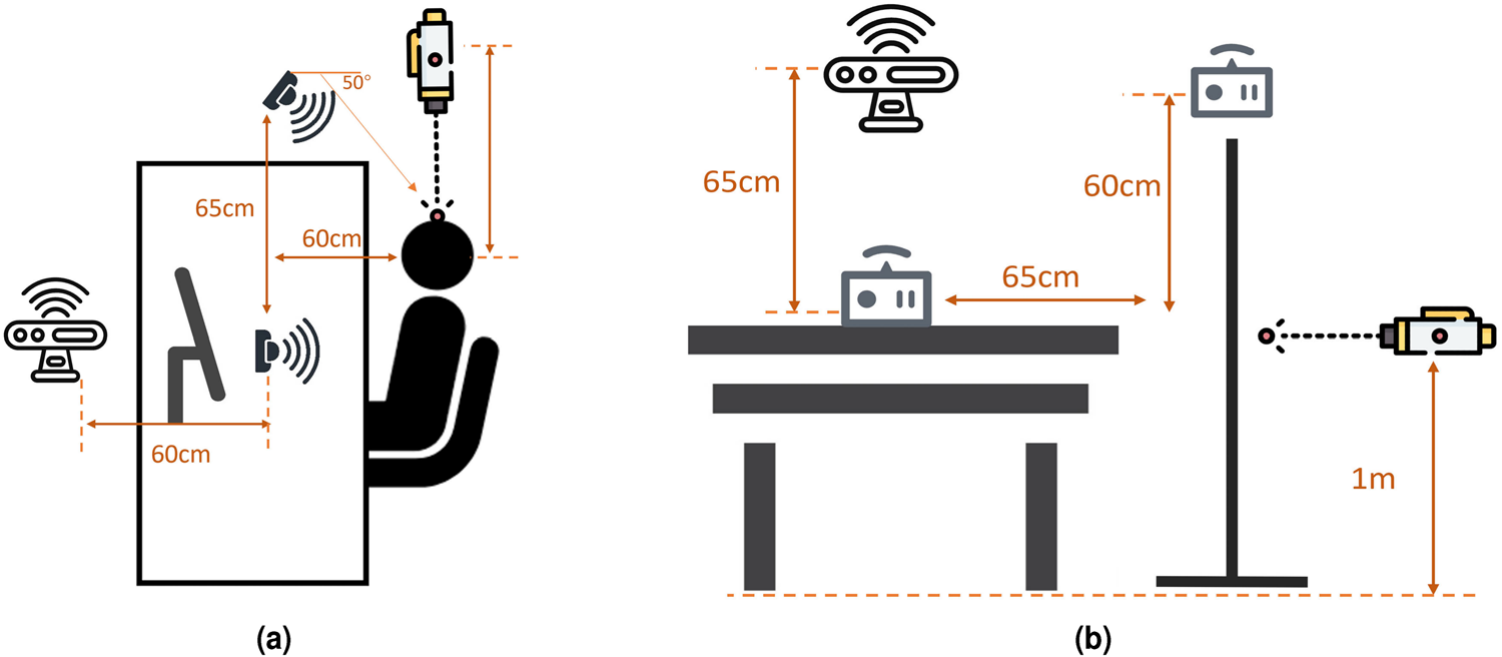
Detailed setup schematic diagram for single person scenario from (**a**) Top view and (**b**) Front view (Laser’s location is not fixed due to the camera based signals process only require the laser directly point to skin of subjects. The UWB radar facing to subject directly was called ‘xe2’ in dataset folder, another is called ‘xe1’).

This section provides the data collection protocol including the introduction of corpus, experiment setup, and data formats. The corpus of the single person scenario is listed in Table 5, and specific setup is illustrated in Fig. 5. During data collection, we asked volunteers to pronounce a specific vowel/word/sentence with timestamps on laptops. All laptops were synchronized using the same NTP server. During collection, volunteers were guided by automatic voice instruction to read the corpus and relax. The timestamps of audio instruction were instantaneously recoreded in kinect/timestamp. However, there is a few seconds of uncontrolled latency in activating all radars and laser equipment, which disrupted the devices’ ability to synchronize acquisition data. In this case, we decided to keep these devices recording for one minute and write timestamp while data collection is activated so that the signals can be cropped according to kinect timestamps. All were recorded alongside the data, which reduces the effort of manually separating the data.

**Table 5:** Corpus list for single subject experiment. The index of each participant is identical to the user label.

Type	Corpus	Index	Participants
vowel	[æ], [i], [ә], [ɔ:], [u:]	1–5 in sequence	User 1–20
word	order, assist, help, ambulance, bleed, fall, shock, medical, sanitize, doctor, accident, rescue, emergency, heart, break	1–15 in sequence	User 1–20
sentences	I need help.	1	User 1–20
	Call for an ambulance.	2	
	The building’s on fire.	3	
	Can you smell smoke.	4	
	Where’s the fire escape.	5	
	There’s been an accident.	6	
	Is there a doctor here?	8	
	The staff sanitized the sickroom.	7	User 1,2,4,6,7
	Medical care is important.	9	
	Don’t worry about bleeding.	10	
	I am having trouble breathing.	7	User 3, 8–13
	I think I’m having a heart attack.	9	
	My heart is failling.	10	
	Need emergency treatment at shock stage.	7	User 5, 14–20
	He need a rescue for a heart attack.	9	
	Don’t worry about falling.	10

**Fig. 6:**
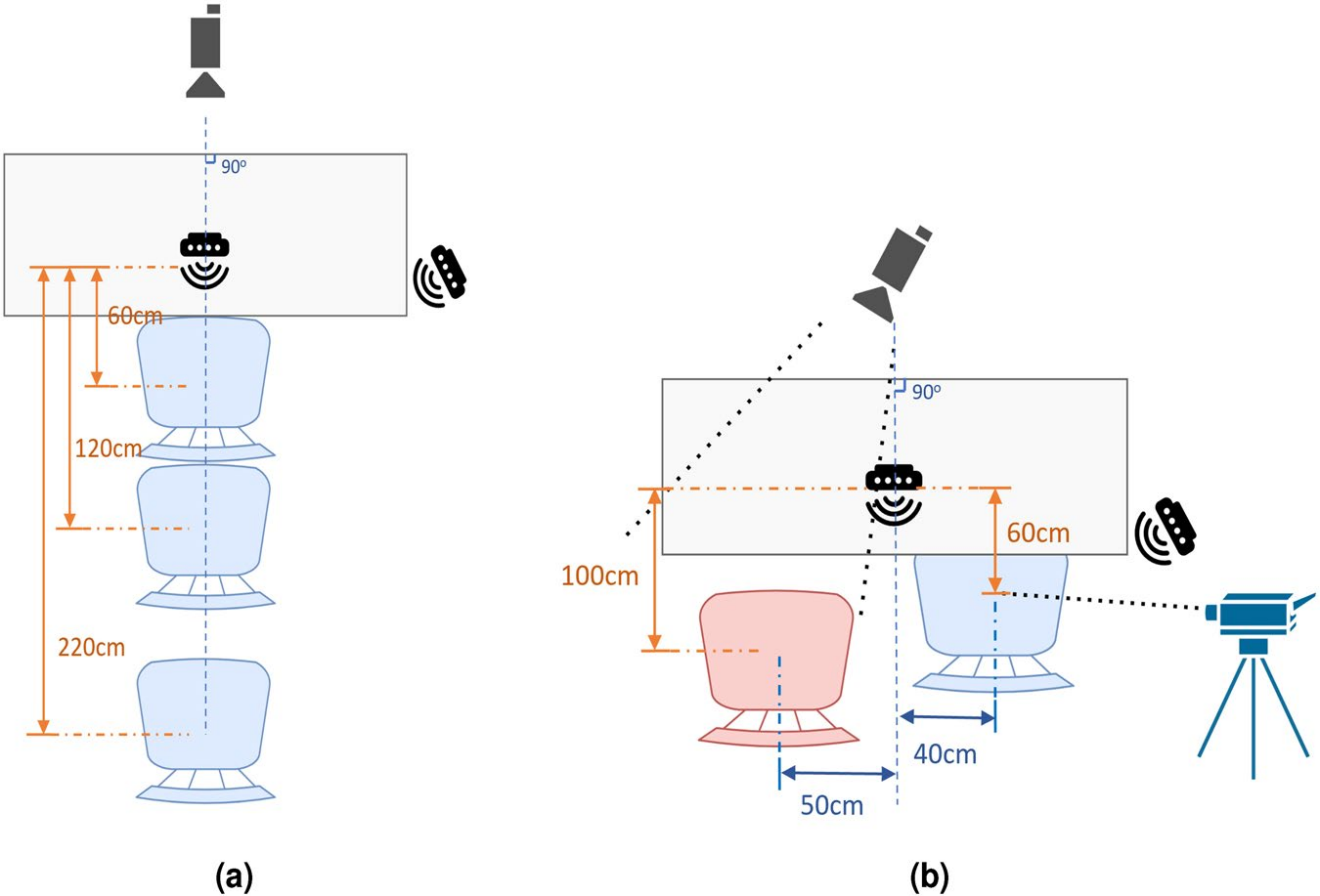
Detailed setup schematic diagram from top view of (**a**) second scenario which considers the distance of 120 cm and 220 cm between sensor and speaker, and (**b**) third scenarios which considers speech data collected under multiple speakers. Camera was set to collect left speaker and laser to right speaker.

Meanwhile, we also collected data via different distances in the single-person scenario and the two-person scenario, which is shown in Fig. 6, with corpus listed in Table 6. Instead of original 60 *cm*, we asked volunteers to sit 1.2 *m* and 2.2 *m* away from radar equipment, respectively, which is a potential for researchers to explore the relationship of radar-based audio detection with distance. In addition, in the two-person experiment, we kept one volunteer sitting in the same place as the single-person scenario, and then let another speaker sit on the left side of the first-mentioned volunteer. The two subjects were asked to normally read different corpus shown in Table 7, which was shown on the screen in one minute, without repeating words. The laser equipment was pointed to the first volunteer, and the kinect camera only took information from another subject. This kind of dataset will contribute to multiple audio source separation.

**Table 6:** Corpus list for supplementary experiments of changing the distance.

Type	Corpus	Label	Participants
vowels	[æ], [i], [ә], [ɔ:], [u:]	v1–5 in sequence	User 4 of 1.2 m and 2.2 m (Index No. 24 and 25 in dataset), User 5 of 1.2 m and 2.2 m (Index No. 26 and 27 in dataset)
words	order, ambulance, medical, sanitize, accident	w1–5 in sequence	
sentences	Call for an ambulance	s1	
	There’s been an accident	s2	
	The staff sanitized the sickroom	s3	
	Is there a doctor here?	s4	
	Medical care is important.	s5

**Table 7:** Corpus list for supplementary experiments of two-person scenario.

Type	Corpus	Label	Participants
article	From view of Kinect, volunteer on the left side read ‘Mr Sticky’, on the right side read ‘The king of the birds’.	b1–11	User 6 (Left) and User 4 (Right), recorded in Index 21
		b12–22	User 4 (Left) and User 6 (Right), recorded in Index 21
		b1–11	User 4 (Left) and User 5 (Right), recorded in Index 22
		b12–23	User 5 (Left) and User 4 (Right), recorded in Index 22
		b1–11	User 5 (Left) and User 1 (Right), recorded in Index 23
		b12–23	User 1 (Left) and User 5 (Right), recorded in Index 23
static	sitting without speaking	b24–26	User 5 (Left) and User 4 (Right), recorded in Index 22

The reading materials are referred from corpus publication^30^.

## Data Record

**Fig. 7:**
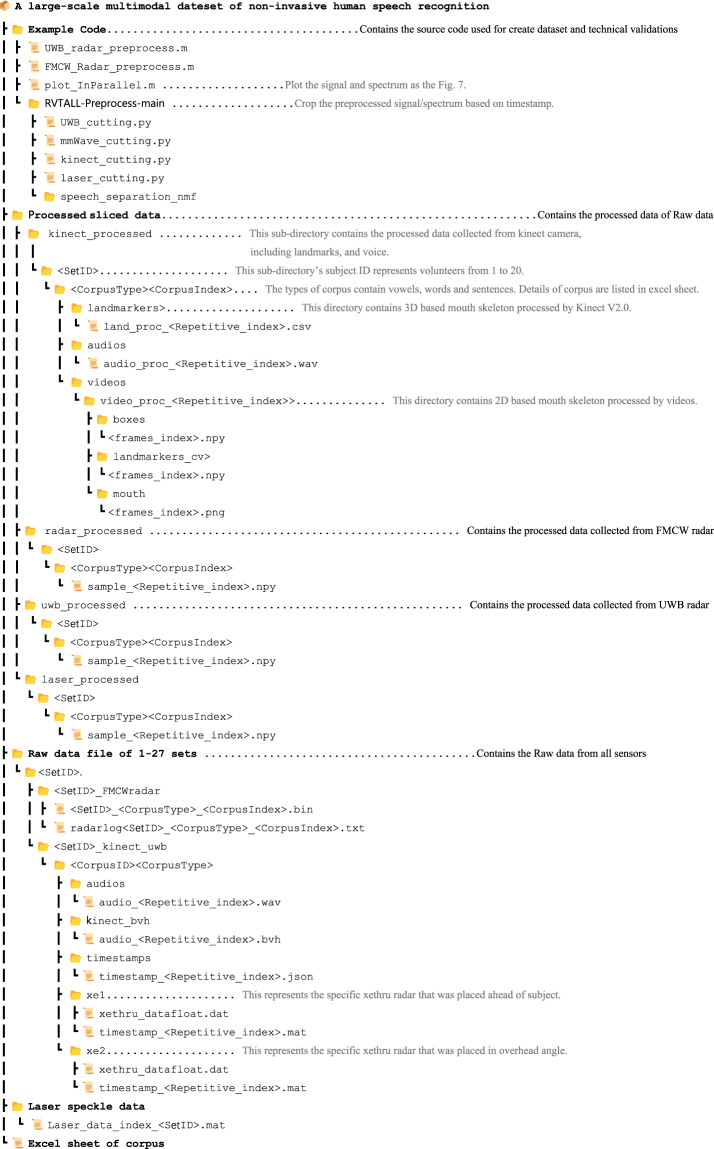
The structure of the multimodal speech dataset.

The dataset is archived within figshare open access repository^17^, designed for ease of access and reproducibility. Detailed in this Fig. 7 is the file structure and content description of the dataset.

### Data storage structure

Processed data files, such as *land_proc_<Repetitive_index>.csv*, include columns for coordinates of detected landmarks by Kinect V2.0, among other variables. The methodology for data collection is expounded of previous sections in this document, providing the necessary context for dataset curation. After saving the data, all files were integrated into specific folders according to the data class, of which structure is illustrated in Fig. 7. The entire dataset was divided into raw data and processed data to match the limitation of file size. Firstly, due to the data size limitation of file, we put mmWave FMCW radar data and laser data in a separate folder and other sensors in another. The radar signals files were kept in binary format with radar timestamps in text format (FMCW radar signals of subject 12 were missing).

Meanwhile, information from kinect and two UWB radars was kept in same folder as the similar storage structure, which contains timestamps in JSON format, audio in WAV, landmarkers of user’s head in BVH, and UWB radar signals in MAT format. Additionally, to ensure a license-free distribution of the dataset, the preprocessed data was converted from MAT to NPY and BVH to CSV files regarding the usages. The description below completely introduces the statement of data files recorded in the proposed dataset.

### Raw data statement

- Comprising raw data from all sensors, segregated by subject IDs. Each subject’s data is further segmented by type of modality, including laser data, FMCW radar signal, UWB radar signal and kinect based audio, facial skeleton and timestamps: <*SetID>_<CorpusType>_<CorpusIndex>.bin*: Raw FMCW radar signals in binary format.
- *radarlog <SetID>_<CorpusType>_<CorpusIndex>.txt*: Raw radar logs with timestamps.
- *laser_data_index_<SetID>.mat*: Laser data matrices with timestamps.
- *timestamp_<Repetitive_index>.json*: Kinect timestamp records including audio and landmark data.
- *audio_<Repetitive_index>.wav*: Original audio recordings.
- *audio_<Repetitive_index>.bvh*: Landmarks of facial expression.
- *xethru_datafloat.dat*: Raw UWB radar signal.
- *timestamp_<Repetitive_index>.mat*: UWB radar timestamps.

### Processed data statement

Each subject’s folder lies processed data in NPY format, with the same name of data files of *sample_<Repetitive_index>.npy* in separate folder, detailed as follows:

- *kinect_processed*: Data from Kinect processed to obtain landmarks of *land_proc_<Repetitive_index>.csv*, vocal features of *audio_proc_<Repetitive_index>.wav* and video frame features of boxes and landmarks of <*frames_index>.npy*, and mouth frame of <*frames_index>.png*.
- *radar_processed*: FMCW radar data tailored for specific signal characteristics.
- *laser_processed*: Laser data processed pertinent to facial recognition tasks.
- *uwb_processed*: UWB radar data tailored for specific signal characteristics.

## Technical Validation

Effectiveness of the collected data is validated in three parts for validation and benchmark: signal processing and analysis, laser signals based speech separation, and multimodal based speech recognition.

### Signals analysis

**Fig. 8:**
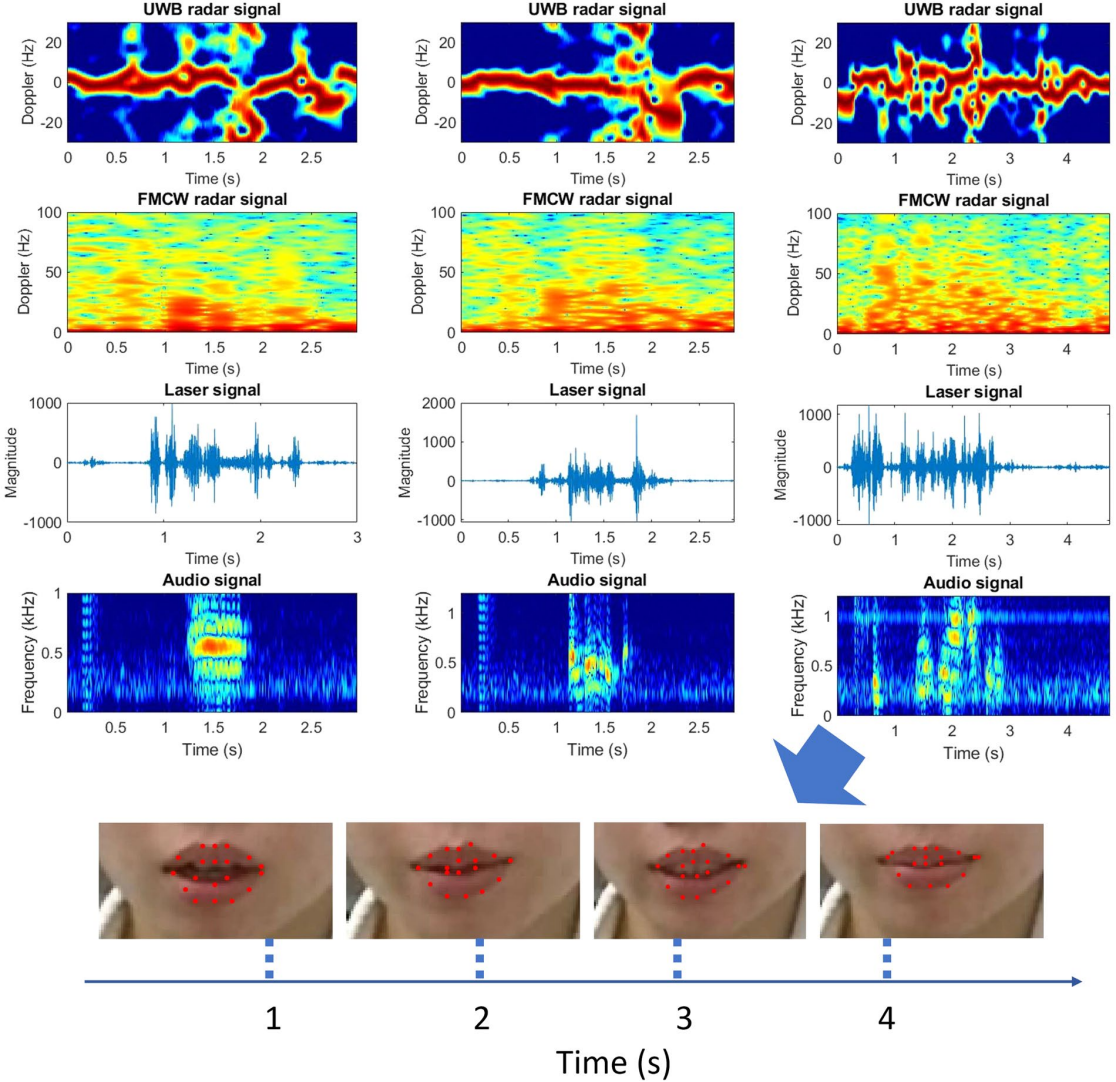
Multimodal data illustration including UWB and mmWave radar signal, laser, audio, image, and mouth skeleton points. From left to right columns, the first represents the volunteer is speaking of vowel ‘[ɔ:]’, second is speaking of word ‘bleed’, and the third is speaking of sentence ‘There’s been an accident’. The last row illustrates the camera vision of the volunteer’s mouth with the processed skeleton.

In this section, we analyze the entire process of lip motion and vibration of the vocal fold combined with video frames of the skeletal mouth and information on the voice, as shown in Fig. 8.

For UWB and FMCW radar signals, we transferred the raw data to the Doppler spectrum, shown with the speech spectrum and skeleton motion. The Fig. 8. shows all synchronized data types that were collected in the dataset. For UWB data, to sanitize the stationary object, the raw signals were first multiplied by a moving target indication (MTI) filter, which is a radar process method that allows the UWB radar to detect and track targets that are moving in relation to the radar devices. From Eq. 1, we know that the channel impulses indicate different ranges. To consider all channel vibrations, we calculate the short-time Fourier transformation (STFT) result on each channel and then add all channels together, which is shown in Fig. 8.

For the FMCW radar, the primary step involves converting the IQ data to range-bin data via 1D-FFT. Notably, our approach emphasizes a distinct form of beamforming using FFT in the AoA (Angle of Arrival) dimension. This FFT-based beamforming is designed to obtain precise angle information, capturing the location of radar signals and thus assisting in the accurate detection of movements, especially in the mouth and vocal folds. Instead of conventional methods that harness the velocity dimension with multiple chirps, our strategy elevates the frame rate to yield refined and continuous radar signals. With the vast capabilities of radars, it’s essential to filter out superfluous data. We employed MTI to sieve out noise echoed by static objects. Subtracting range-angle spectrograms at certain intervals aids in diminishing false alarms due to substantial indoor clutter, optimizing the clarity of our results. Consequently, by focusing on the radar strength within our range of interest—which corresponds to the human’s location, we could discern detailed information about mouth movement and vocal fold vibrations^18^. Given the predetermined speaker location, the IQ signal regarding that specific range-angle bin can be extracted and analyzed^19^. As the benchmark validation, we only extract the amplitude variation of radar signal.

Furthermore, we transferred the video to images with 30 frames per second and voice signals in spectrum, shown in Fig. 8 together with Doppler spectrograms of UWB and FMCW radar signals. To retrieve the sound signals from raw laser speckle data, an optical flow-based method, notably the Farneback algorithm, is utilized to estimate the displacement of laser speckles on participants’ faces. The input of this algorithm is every frame, denoted as a 2D function (f (x, y)), whereas a quadratic polynomial expansion is adopted to approximate the gray value of each pixel and its neighbors. The signals model based on the local coordinates of the selected pixel could be written as the Eq. 2.

2 $$f(x)={x}^{T}Mx+{n}^{T}+q$$

where x is the local coordinate (*x*, *y*), *M* is a symmetric matrix equal to $$\left[\begin{array}{cc}{C}_{4} & {C}_{6}/2\\ {C}_{6}/2 & {C}_{5}\end{array}\right]$$, n is a vector equal to $$\left[\begin{array}{c}{C}_{2}\\ {C}_{3}\end{array}\right]$$ and q is a scalar equal to *C*_1_, *C*_1_ to *C*_6_ are the coefficients of the quadratic polynomial expansion. The new signals could be expressed using a displacement index Δ*d* as the Eq. 3 indicates.

3 $$f(x-\Delta d)={(x-\Delta d)}^{T}{M}_{1}(x-\Delta d)+{n}_{1}^{T}(x-\Delta d)+{q}_{1}\quad \quad \quad g(x)={x}^{T}{M}_{2}x+{n}_{2}^{T}+{q}_{2}$$

Simply let $f(x-\Delta d)=g(x)$, then we can get ${n}_{2}={n}_{1}-2{M}_{1}\Delta d$, leading to the solution of displacement index. Then the computed optical flow needs to be filtered with a band-pass filter. The cut-off frequency of the filter is chosen to be 80 *Hz* and 255 *Hz* for removing the frequency components caused by non-speaking activities such as head and skin movement. Then we integrate all sorts of cropped data that were mentioned above and show the matched samples in Fig. 8.

### Speech separation task assisted by laser signal

Speech separation is used in a variety of applications, including telecommunications, hearing aids, speech recognition systems, and audio and video conferencing systems. These techniques typically involve analyzing speech signals from different sources and then using filtering, spectral shaping, or other signals processing methods to remove or reduce unwanted components while preserving or enhancing the speech signal. Since the components of the human voice are mainly in the same frequency band, the frequencies of the different components are mixed together. Therefore, identification in the frequency domain alone is almost impossible.

**Fig. 9:**
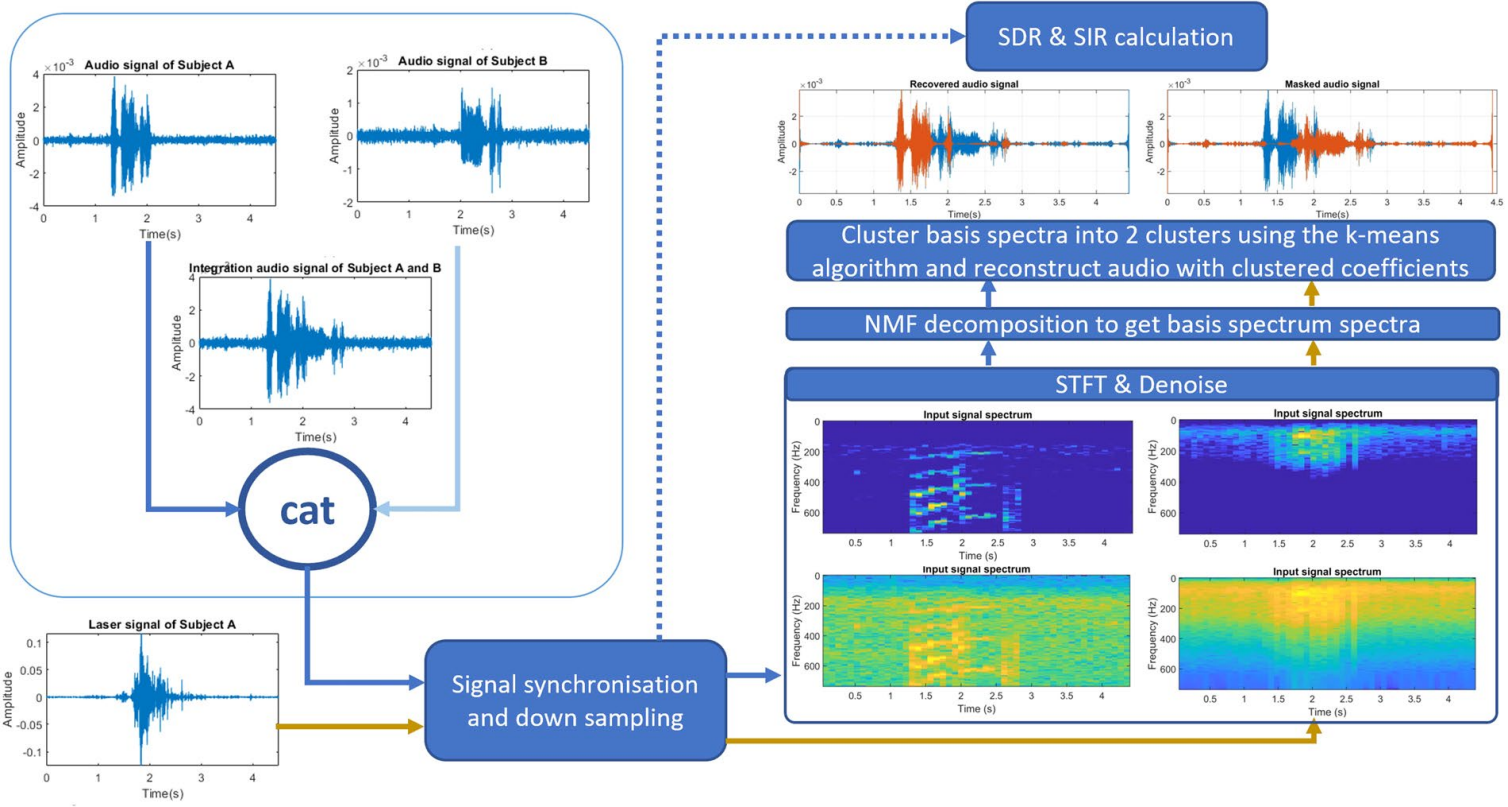
Speech separation scheme of mixed audio signals and laser-speckle signals using a NMF based method.

Laser speckle signals are able to observe the vibration of skin covering cheek through capturing the laser speckle motion. We empirically observe that there is a relationship between laser signals and audio signals. Previous work also approved that laser speckle signals can be used for audio denoising^20^, which can be replicated with our dataset. Meanwhile, in our dataset, we introduce the laser speckle signals as the source of speech enhancement/separation in single channel case. As the validation example of speech enhancement application with laser speckle signal, we will introduce non-negative matrix factorization (NMF) based work which utilizes the processed laser signals to enhance the voice made from the speaker. Then the rest of the audio components of another speaker can be revealed. Validation experiments follow the scheme shown in Fig. 9. The input contains audio and laser signals from subject A, and delayed audio signals from subject B. First, we add up two audio input to simulate the complex environment of two volunteers speaking simultaneously. Then the laser and audio signals are synchronized with timestamp and frequency. Meanwhile, the audio signals were downsampled to match the vibration frequency limit of the vocal folds. After that, the applied short time fourier transformation with hanning window to get spectrogram of laser and audio signal. Then we applied NMF to the magnitude spectrogram to obtain a set of basis matrix and corresponding activation coefficients for each source. The decomposed basis matrices reflect the speech characteristic in the time domain. Two clusters of matrices can be separated depending on whether they are correlated with reference laser signals. Finally, we reconstruct the audio with time-frequency mask that was clustered. The detailed steps can be referred to the code with the dataset.

To explore the performance of our scheme, we selected the collected laser-audio dataset of sentences from No.17 and 18 subjects. We named the audio signals of laser target as recovered audio signals, and another as masked signals. The average signal-to-distortion ratio (SDR) and the signal-to-interference ratio (SIR) of recovered signals are 1.04 dB and 35.97 dB respectively. Meanwhile, the performance of masked signals that were not correlated with laser signals, gains 0.0002 dB of SDR and 35.97 dB of SIR. Overall, this work provides another view of single channel based speech enhancement and separation tasks. Meanwhile, the data collected from third scenario provides raw signals under two volunteers’ speech, which is a good source for users to test their methods.

### Multimodal speech recognition

**Fig. 10:**
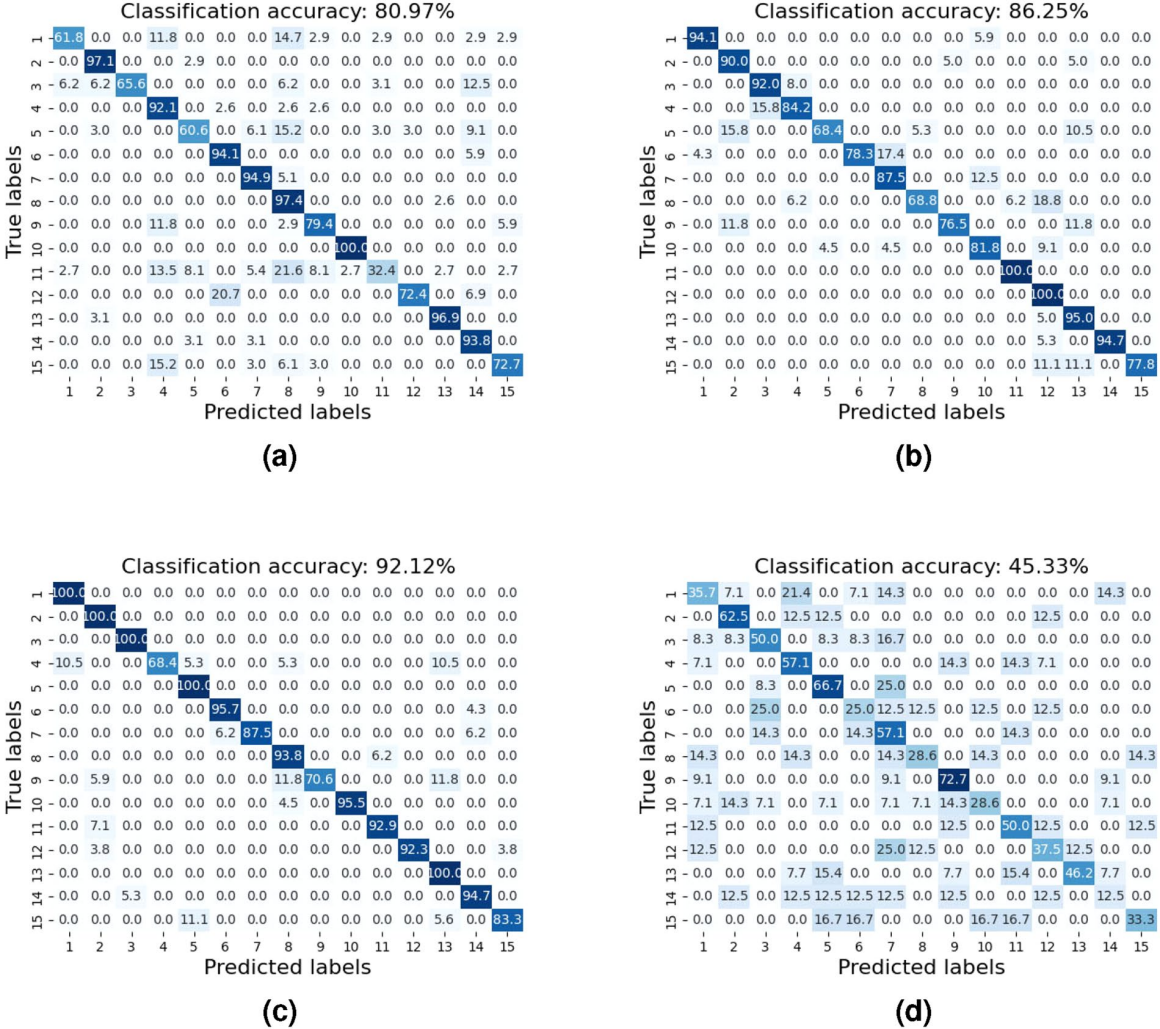
Classification performance of human speech across 15 Words with confusion matrix of (**a**) UWB radar, (**b**) video stream, (**c**) audio signal, (**d**) laser speckle signals.

For the benchmark of speech contents classification, we selected UWB radar, laser, audio and video data of 5 subjects and established a CNN-based ResNet classification network. The batch size is 16 and the primary learning rate is 0.01. Each model uses 80% data for training and 20% for validation and is trained for 50 to 100 epochs. The performance is shown in Fig. 10. It is evident that there is potential to improve speech recognition performance based on non-invasive UWB and laser technology.

**Fig. 11:**
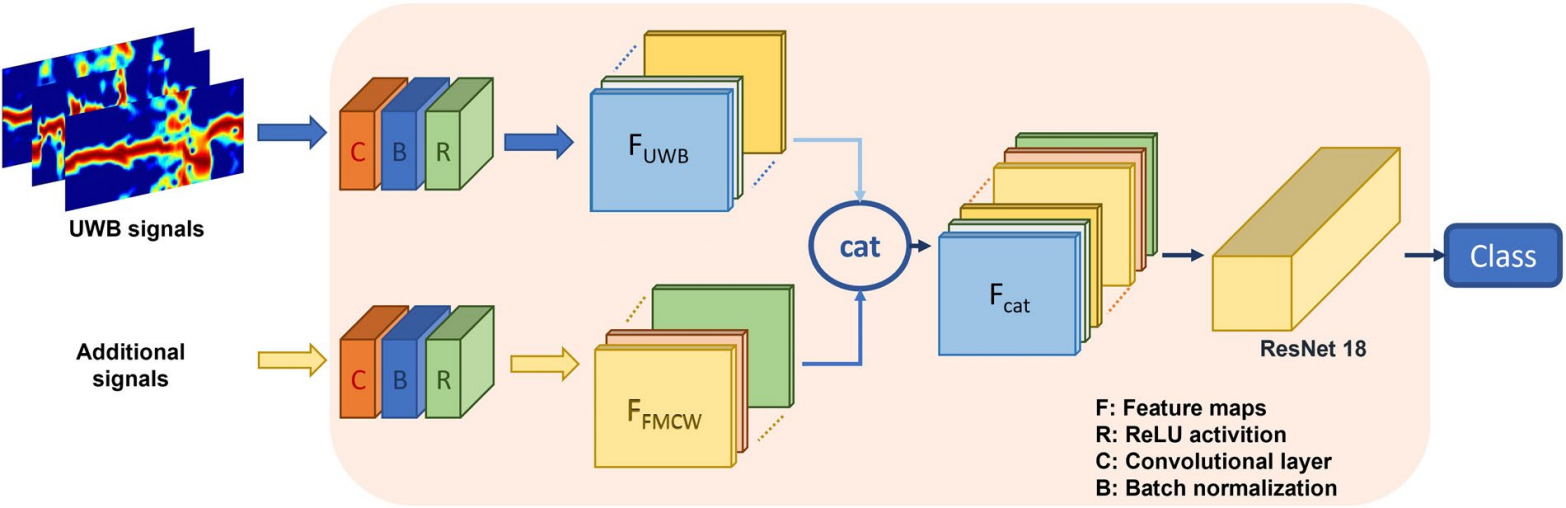
Multi-modal sensor fusion scheme for word speech classification.

**Fig. 12:**
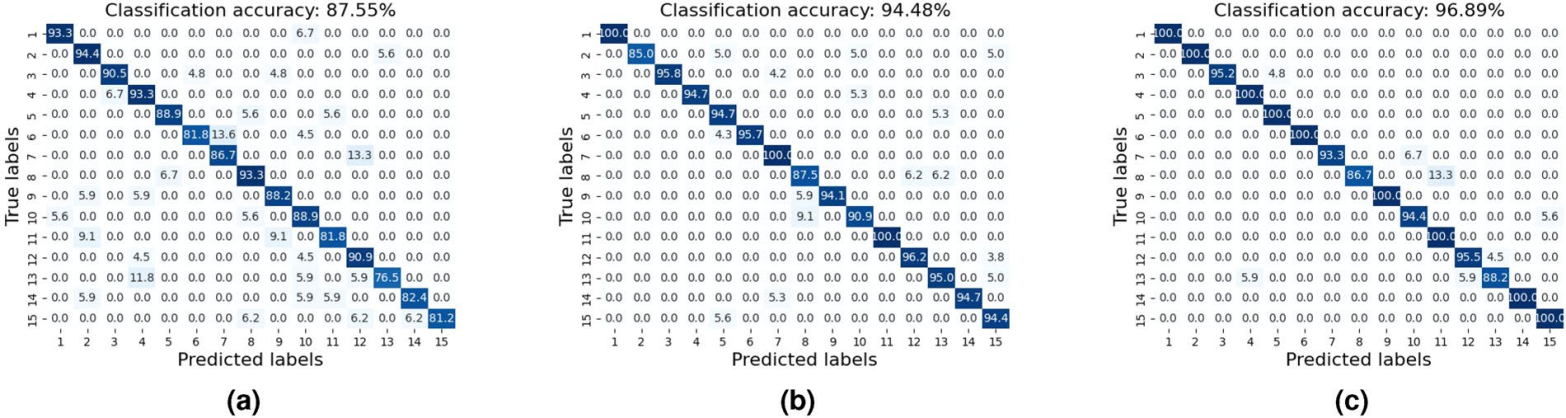
Classification performance of human speech across 15 Words with confusion matrix of (**a**) the fusion of video and UWB, (**b**) the fusion of audio and UWB, (**c**) the fusion of video, audio and UWB.

**Fig. 13:**
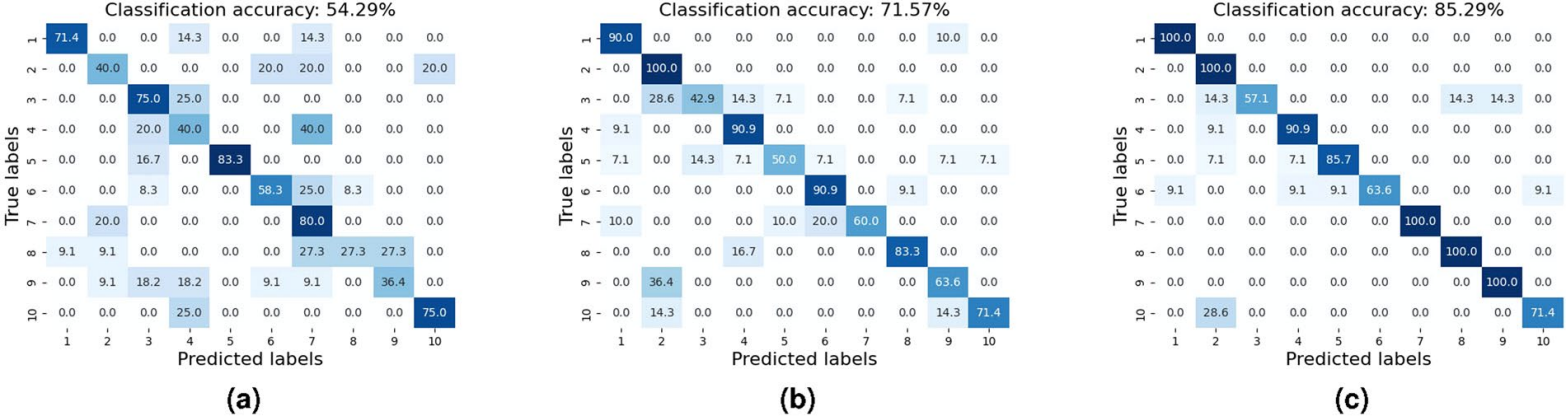
Classification performance of human speech across 10 Sentences with confusion matrix of (**a**) UWB signals, (**b**) the fusion of video and UWB, (**c**) the fusion of audio and UWB.

Besides, we further considered a sensor fusion scheme that combines data from UWB radar and multiple sources for word recognition. We employed a multi-input ResNet18 for this task, which includes two input blocks consisting of a convolutional layer, a batch normalization layer, and a ReLU activation, as shown in Fig. 11. The initial feature extraction was completed by feeding spectrograms from the UWB radar and additional information into their respective input blocks. The resulting feature maps were then stacked along the channel axis and processed by ResNet18 for final analysis. Then, we also applied audio data on word recognition as comparison with radio-based methods. The performance of the multimodal based recognition systems is shown in Fig. 12. Meanwhile, we adopt the methods on sentences classification with the result shown in Fig. 13.

**Fig. 14:**
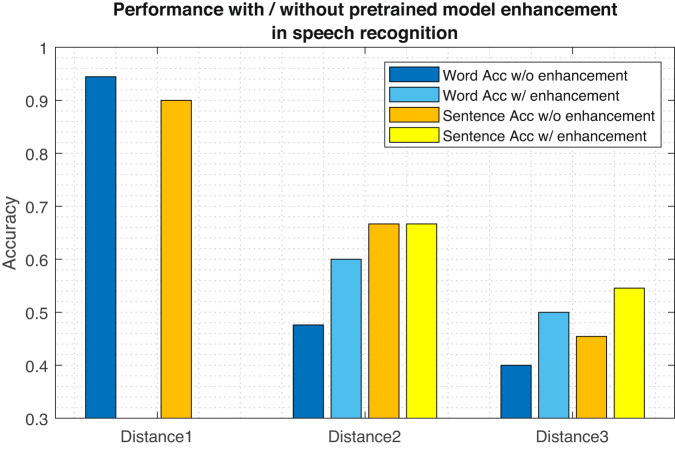
Comparative performance with or without pretrained model enhancement in speech recognition.

Another challenge encountered in real-world applications is the varied “context”. For instance, in our scenarios, the dynamic distance between a target and the receiver may cause a substantial performance decline for radar-based models trained on a fixed-distance dataset. However, obtaining new data samples to account for different “contexts” is often impractical. Consequently, we further explore the use of our dataset in transfer learning tasks, with the goal of enhancing the scalability of radar-based lip-reading systems. By fine-tuning a pre-trained model with minimal additional data, we strive to address the challenges posed by diverse contexts. We carried out experiments using UWB signals gathered from volunteer 4 at three distinct distances. The pre-training phase was conducted on data from the first distance, employing a batch size of 16, a learning rate of 0.01, and 50 to 100 training epochs. Subsequently, we assessed the model’s performance at the second and third distances, both with and without fine-tuning (utilizing the same training settings as the pre-training phase), focusing on its SSR performance. A comparison of sentence and word performance revealed that complex sentences presented greater challenges for SSR with radar. Additionally, we examined the impact of varying distances between the user and sensors in Fig. 14.

## Usage Notes

The multimodal speech detection dataset is accessible with the doi link of the dataset^17^. Data users are encouraged to analyze the data with the example script provided with the dataset. The functionalities of the script are described in the following section. It is imperative to underscore that the processing structure we present serves as a foundational method. We anticipate and encourage readers to devise their own methodologies using the dataset, tailored to specific targets. We would like to recommend that the user who wants to process their own algorithm on raw data, pay attention to the synchronization among multiple sources with provided timestamps. All timestamp files of each sensor are listed in Fig. 7. To reduce the potential timestamp mismatch, our approach is to record the activation timestamp of each sensor meticulously. This timestamp is then used as a reference to calculate the timestamp for each frame of data. Meanwhile, we created a github Repo to update the example processing code with dataset: https://github.com/G-Bob/Multimodal-dataset-for-human-speech-recognition. Finally, 7-ZIP was used to compress all the subfolders depicted in Fig. 7, making them easily downloadable for users.

## Data Availability

Matlab and Python scripts are provided in the codes directory of dataset for the users to replicate some of the figures: • *FMCW_Radar_process.m* This script is used to load the raw signals recorded by the AWR2243 radar. Then it is used to visualise the first and second FFT through distance dimension and angle dimension, respectively. Lastly, by reading the human location’s phase variation, we can get human-related signals, including lip motion. This step can be transferred to PYTHON and other coding methods which supports reading binary files. • *UWB_radar_process.m* This script is used to load the raw signals recorded by the Xethru X4M03 radar and process the data to STFT spectrums. This step can be transferred to PYTHON and other coding methods which supports reading binary files. • *plot_InParallel.m* This script provides a template to plot the spectrums that are shown on paper. First, the preprocessing data is needed to be downloaded. • *uwb_cutting.py; mmWave_cutting.py; kinect_cutting.py; laser_cutting.py;* This script can be utilised to cut the different data sequence in NPY format with given Kinect timestamp, and convert BVH to CSV files. In advance of this step, the radar signals should be processed to spectrums with provided raw data in DAT. This step can be directly used with the radar scripts we provide.
